# Privacy of Study Participants in Open-access Health and Demographic Surveillance System Data: Requirements Analysis for Data Anonymization

**DOI:** 10.2196/34472

**Published:** 2022-09-02

**Authors:** Matthias Templ, Chifundo Kanjala, Inken Siems

**Affiliations:** 1 Institute of Data Analysis and Process Design Zurich University of Applied Sciences Winterthur Switzerland; 2 Department of Population Health London School of Hygiene and Tropical Medicine Lilongwe Malawi; 3 Economics and Social Statistics University of Trier Trier Germany

**Keywords:** longitudinal data and event history data, low- and middle-income countries, LMIC, anonymization, health and demographic surveillance system

## Abstract

**Background:**

Data anonymization and sharing have become popular topics for individuals, organizations, and countries worldwide. Open-access sharing of anonymized data containing sensitive information about individuals makes the most sense whenever the utility of the data can be preserved and the risk of disclosure can be kept below acceptable levels. In this case, researchers can use the data without access restrictions and limitations.

**Objective:**

This study aimed to highlight the requirements and possible solutions for sharing health surveillance event history data. The challenges lie in the anonymization of multiple event dates and time-varying variables.

**Methods:**

A sequential approach that adds noise to event dates is proposed. This approach maintains the event order and preserves the average time between events. In addition, a nosy neighbor distance-based matching approach to estimate the risk is proposed. Regarding the key variables that change over time, such as educational level or occupation, we make 2 proposals: one based on limiting the intermediate statuses of the individual and the other to achieve k-anonymity in subsets of the data. The proposed approaches were applied to the Karonga health and demographic surveillance system (HDSS) core residency data set, which contains longitudinal data from 1995 to the end of 2016 and includes 280,381 events with time-varying socioeconomic variables and demographic information.

**Results:**

An anonymized version of the event history data, including longitudinal information on individuals over time, with high data utility, was created.

**Conclusions:**

The proposed anonymization of event history data comprising static and time-varying variables applied to HDSS data led to acceptable disclosure risk, preserved utility, and being sharable as public use data. It was found that high utility was achieved, even with the highest level of noise added to the core event dates. The details are important to ensure consistency or credibility. Importantly, the sequential noise addition approach presented in this study does not only maintain the event order recorded in the original data but also maintains the time between events. We proposed an approach that preserves the data utility well but limits the number of response categories for the time-varying variables. Furthermore, using distance-based neighborhood matching, we simulated an attack under a nosy neighbor situation and by using a worst-case scenario where attackers have full information on the original data. We showed that the disclosure risk is very low, even when assuming that the attacker’s database and information are optimal. The HDSS and medical science research communities in low- and middle-income country settings will be the primary beneficiaries of the results and methods presented in this paper; however, the results will be useful for anyone working on anonymizing longitudinal event history data with time-varying variables for the purposes of sharing.

## Introduction

### Background

Although health research data sharing has many benefits and great value [1,2], one of the main concerns is maintaining the privacy of study participants. The rationale for both data sharing and privacy is widely recognized. In the field of medical science research, the issue of privacy is central to good ethical practice. Anonymization of data provides an opportunity to mitigate this tension between sharing data and preserving the privacy of those whose data are shared. However, it is often unclear how data can be shared without unduly compromising the privacy of the individuals included in a data set.

A fundamental issue with personal data disclosure is whether an attacker can learn anything about an individual if the data or analysis results are provided or predictions are made. On the one hand, one can ask whether an attacker can successfully match individuals with the data at their disposal. In addition, are attackers’ efforts (and related costs) higher than the benefits of disclosing information? On the other hand, the needs of the users of data are of high utility, allowing for high-quality analysis. Data providers are interested in providing such information without disclosing the identities of the individuals in the data.

Similar to all other areas of health research, longitudinal population studies in low- and middle-income countries (LMIC), such as health and demographic surveillance system (HDSS) [3], face the challenge of finding the right balance between data sharing and privacy protection.

The HDSS must take a position that allows the sharing required by research funders and journal publishers [2,4] while minimizing the risk of compromising the privacy of individuals who make their data available for research.

However, the important issue of health data privacy has not been adequately explored in LMIC in general and HDSSs in particular. HDSSs currently share data in most cases without anonymizing them beyond masking direct identifiers [5]. There is a possibility that attackers may use indirect identifiers such as education level, sex, and age—in cases where these are shared [6]—to identify participants and, consequently, their health status, which they did not intend to share beyond the boundaries of the research in which they participated. The extent of such risks has not been fully explored in the HDSS data sets, and consequently, no measures have been taken to mitigate these risks; that is, to the best of our knowledge, this has not been addressed in the literature on health, statistics, and privacy.

Note that for some selected data sets and general anonymization problems, the World Bank Group, PARIS21 and Organization for Economic Cooperation and Development, and the International Household Survey Network supported the development of the anonymization software sdcMicro [7], and they all recommend it [8]. sdcMicro is actively used in many organizations, ranging from statistical offices [9] and social and political science [10] to the United Nations High Commissioner for Refugees [11] and health [12-14]. However, there is a need to justify the use of this software for the specific needs arising from longitudinal population health data in LMIC.

Longitudinal data include records of different attributes of the same participants observed and measured at multiple points in time. Existing theories and software are suitable only for anonymizing and assessing the disclosure risk of cross-sectional data. An extension of this theory is needed to quantify and control the disclosure risk for longitudinal data.

### Karonga HDSS

An HDSS is a combination of field and computing procedures for collecting demographic, health risk, and exposure and outcome data from a defined population within a defined geographical area on a longitudinal basis [3,15]. HDSSs are set up to monitor open or dynamic population cohorts, building longitudinal databases of this population over time [15]. A substantial body of literature has considered various HDSS aspects, including the rationale for their establishment in LMIC [3,15], the definition of core HDSS concepts and processes [5,16], and the reference data model [17] among many others. The data set used for illustration is from an HDSS in Malawi, the Karonga HDSS. This HDSS has been described in detail elsewhere [18]. Briefly, its surveillance site is in northern rural Malawi and has been in operation from its initial census in 2002 to 2004. The Karonga HDSS contains longitudinally linked health data from the study population.

The Karonga HDSS is part of a collaborative research program under the Malawi Epidemiology and Intervention Research Unit [19].

### HDSS Core Residency Data

The generic data set structure on which we based this data anonymization requirements analysis is in the core residency data format. This standard data set is widely used in HDSS for data sharing and analysis [19]. An extended version of this data set is comprehensive enough to cover the considerations that need to be made in anonymizing HDSS event history data. This data set essentially comprises the core HDSS events for each individual under surveillance and attributes relating to the individual and to the core events. The events occur in a particular order that defines entry or exit from the study population. The first event for any individual is one of the following: a baseline census enumeration, a birth, or an in-migration. The last event is one of the following: an out-migration, a death, or the end of observation (censoring). The intervening events observed for any individual need to be logical; for example, an individual born within the surveillance area cannot have in-migration as the next event. The core events change the residency status of an individual and, thus, the name of the data set, core residency data [20].

The basic form of the core residency data includes the following variables: an individual identifier, date of birth, sex, core event, and event date. This form contains all the data on the numerators and person-years of surveillance (exposure) required to calculate the demographic rates for the HDSS population and perform event history analyses.

This basic form can be extended to capture other observations made within the HDSS population. These may include disaggregation of the migration events by distinguishing between migration within the surveillance area (internal) and migration to or from outside the area (external), as well as the inclusion of attributes that change over time, such as education level, occupation, and specific disease status (eg, HIV and tuberculosis).

To elaborate on the anonymization requirements, we distinguish between three variable groupings that can go into these HDSS core residency data:

1. Static variables: These are variables in which the observations on individuals do not change over time, such as sex and date of birth.
2. Status (time-varying) variables: These are variables in which the observations on individuals change over time, such as occupation or education level.
3. Core events variables: These are the variables in which the observations are specific to the event. The observed event and the event date fall into this category.

Our approach investigates the requirements for anonymizing variables falling into these 3 groups.

### Karonga Residency Data

The variables in this data set largely overlap with those found in the publicly available Karonga HDSS core residency data set on the iSHARE data repository [21]. The extended version used in this study has status variables on occupation and education level, in addition to those found in the Karonga core residency file.

This data set contains information recorded from October 1995 to the end of 2016, comprising 14 variables, 280,381 rows (events), and 72,935 individuals ever observed since the HDSS’s inception.

The main variables of the data set for this work are as follows:

- Static variables: sex
- Status variables: occupation with categories not working, student, unskilled manual, farmer, fisherman, skilled manual, nonmanual, small trader or business, unskilled manual, skilled manual, nonmanual, and professional; and education with categories none, 1 to 3 years primary, 4 to 7 years primary, primary completed, Junior Certificate of Education completed, Malawi School Certificate of Education completed, and tertiary
- Core event variables: event code with dates on the baseline, date of birth, in-migration, out-migration, and date of death
- Household ID, mother’s ID, father’s ID, and polygamy ID

### Objective

To contribute toward filling this gap, we propose a set of requirements for anonymizing the HDSS longitudinal data. Our proposal customizes and applies traditional methods that work on the premise of keeping the data quality as high as possible while slightly altering the data until the disclosure risk is below a fixed threshold. The main contributions of this study are as follows:

- We define anonymization requirements peculiar to longitudinal event history data.
- We propose steps to take to meet these requirements, including assessing and controlling for disclosure risk for the static and time-varying variables and core event dates.
- We implement the proposed steps and show the results.
- We place our proposal within the larger context of data anonymization approaches, outlining how our method of choice contrasts with the alternatives within the LMIC HDSS context.

## Methods

In this section, we outline the methods and procedures for anonymizing HDSS core residency data.

### Different Concepts for Different Needs

Our approach of keeping data quality as high as possible by modifying data slightly until the disclosure risk is below a certain threshold does not stand alone but rather is part of a broader ecosystem of data anonymization methods. We briefly review this ecosystem and emphasize that the choice of anonymization approaches depends heavily on the needs of the user group and the cost of implementing the solution. We briefly outline 4 important anonymization concepts before discussing their applicability for sharing HDSS data. They are listed in ascending order of data analysis potential as follows: privacy-preserving computation, synthetic data, secure laboratories, and the approach used in this study (anonymized individual-level data using methods of statistical disclosure control [SDC]). With privacy-preserving computation, data remain on the data owner’s side. This can be extended to a secure multiparty computation with multiple clients (data holders). Two popular privacy-preserving computation methods are differential privacy [22] and federated learning with Private Aggregation of Teacher Ensembles [23]. However, there are several limitations, as highlighted in the studies by Domingo-Ferrer et al [24], Francis et al [25], and Bambauer et al [26]. Furthermore, the user must trust the predictions without evaluating the model and the data behind the model. Another way of providing anonymized data is by generating synthetic data that exhibit the same characteristics as the original data [27], usually using machine learning and statistical modeling methods. Synthetic data typically have very low disclosure but have also relatively low data utility when the original data possess complex structures [6]. Synthetic data can also be used in remote execution environments, whereby registered researchers work on the synthetic data to develop an analysis code, and the staff of the data holder finally runs the code on the original data. The final analysis output is checked for privacy by laboratory staff as this checking can hardly be fully automated [28-30].

### Difficulties in Using Alternative Concepts

For HDSS data, using privacy-preserving computation would mean first setting up a framework to compute privacy and, for known users (test data), providing a predictive value for a meaningful piece of information (eg, the date of migration or the health status of a person) based on a machine learning prediction approach. It is evident that these approaches have some difficulties in providing good predictions for complex longitudinal data sets. Privacy-preserving computational approaches are also not sustainable options for health and survival data for LMIC because of the high cost and the users’ need for detailed data, instead of simply receiving predictions for sensitive information or working with aggregated data. Synthetic, close-to-reality data have the potential of being a viable approach; however, the complexity of longitudinal event history data from HDSS makes it difficult to model and represent all relationships and logical conditions adequately. Remote access to secure laboratories offers the advantage of working on real data but can only provide access to a small number of trusted researchers and requires permanent staff to perform output checks to keep the software on the servers up to date and the server and access secure.

### Methods for SDC

For these reasons, methods of SDC are the most suitable. The core concept of SDC comprises transforming data in such a way as to reduce the reidentification risks of the persons represented in the data. More precisely, the aim of SDC is to reduce the risk to a level below a predefined threshold on the one hand and to maintain the data quality and analysis potential and research questions on the other. This is a complex task that requires the application and development of complex methods and, in our particular case, the understanding of specific health population data sets.

### Data Release Types: Public Use Versus Scientific Use Files

In line with lowering the barriers to data access, as encouraged by funders [2], and in the interest of implementing sustainable data sharing models, open data through the sharing of the so-called public use files [31] would be a typical mechanism for sharing HDSS data. Public use files require that a potential user agrees to the terms of use and then get access to the data without seeking approval from the data custodians. A reason for this is the resource-efficient publication and distribution of data. Once distributed, there is no need for further labor-intensive steps, as is the case with remote execution and remote access solutions. The next level up would be the scientific use files [31]. This requires a potential user to go through a review process by a data access team to confirm that they are a bona fide researcher from a reputable institution. This sharing demands that the custodians set aside staff time to review data access applications, prepare the data for sharing, customize the shared data to suit the request, and communicate and supervise the researchers. These demands of staff time are suboptimal as they will take staff away from their daily work and are rarely sufficiently funded in LMIC medical science research projects.

### Pseudoanonymization

In pseudoanonymization, a string—the exact name of a person or any other direct identification feature (eg, social security number)—is replaced by a pseudonym, usually a 256-bit hash code produced by a cryptography hash function from a salted string [32,33]. The pseudoanonymization of the HDSS core residency data on the iSHARE data repository is performed in a simplified manner. An ascending ID is assigned per person instead of listing their names or identifiers used in the dynamic HDSS databases. Note that as more data with complex interrelationships are shared through platforms such as the Implementation Network for Sharing Population Information from Research Entities (INSPIRE) data, more elaborate pseudoanonymization will become necessary. However, pseudoanonymization does not solve the data protection problem as it only prevents attacks on direct identifiers.

### Identifying Key Variables—the Disclosure Scenario

The key question here is what information does an attacker have access to that they could match with the data to be released to identify individuals? Before the key variables (also often called quasi-identifiers) are identified, a check is made to see what other existing data a potential attacker could access and use to link to the current data and identify individuals. This is called the (archive) disclosure scenario [34]. Existing data may include census, voters’ roll, population surveys, or administrative data held by government departments and national statistical offices. In most LMIC, not many data sets are available for broad access, and hence, this should not be a major problem.

The biggest challenge may be that an attacker has additional knowledge of some information pertaining to an individual in the data being released. This is often called the nosy neighbor scenario in the literature [34]. An attacker can potentially use this information to identify individuals.

In general, defining these scenarios requires input from subject matter experts who work with the data being released and who are also aware of other common data.

### Anonymization Methods for Static and Status Variables

Traditional anonymization of population data uses the concept of uniqueness. By combining several variables (quasi-identifiers from the *Identifying Key Variables—the Disclosure Scenario* section), an individual can be uniquely identified in the data. A key is unique if its frequency is 1, and thus, only one person has the combination of characteristics defined by the key. For example, the key postcode *8404*, citizenship *Austria*, sex *male*, and age *45* are unique in a demographic population data set of Switzerland. A commonly used concept for measuring uniqueness and “almost uniques” is k-anonymity. A data set is k-anonymous if each key (ie, combination of key variables) belongs to at least *k* observations. An approach that also evaluates subsets of key variables is called the special uniques detection algorithm [35,36]. This approach allows for a more detailed analysis and evaluation of uniques in subsets of key variables.

To achieve k-anonymity and low special uniques detection algorithm scores, the first step typically involves use case–specific recoding of the categorical key variables into broader categories [6]. With recoding, the risk can be significantly reduced. If some individuals still have an increased risk and further recoding would lead to an excessive loss of quality of data, local suppression is typically considered next [6]. This suppresses certain values to guarantee, for example, k-anonymity. The aim is to find specific patterns in categorical key variables and replace these patterns with missing values. (heuristic) optimization methods must be applied to find a minimal suppression pattern [7].

If the number of categorical key variables is large or many of these variables have many categories, the number of keys in a data set is large, and many keys will be unique. In this case, recoding and local suppression would significantly change the data to achieve, for example, k-anonymity. Applying the postrandomization method (PRAM) [37] to a subset of key variables would be a good alternative to recoding and suppressing all key variables. In the PRAM, values are exchanged between the categories of a variable with certain transition probabilities. An attacker can never be sure whether a value is true or has been swapped.

### Handling Static and Status Variables With Varying Status of a Person Over Time

Cross-sectional data sets typically contain observations for a single time point, and the application of anonymization methods is generally straightforward (eg, using the guidelines presented by Templ et al [6]).

In the following paragraphs, the extension to longitudinal information, in particular to status variables (eg, *occupation* or *education*), for which the observed values (can) change over time, is discussed. Table 1 shows the problem of using a toy data set with 2 individuals in a simplified manner. It can be easily seen that for person 1, both educational level and occupational have improved over time. When only the baseline status in 2010 is considered, both individuals share the same level of education and occupation category; thus, they are not unique in the data set. If only 2015 were considered, the 2 individuals would not be unique. If only the latest status of a person is considered, both individuals would be unique in this toy data set, considering the key variables of occupation and education level. Moreover, if each status is reported each year, the 2 individuals would also be unique.

A number of alternative representations could be used to anonymize the status variables, each of which has its own advantages and disadvantages.

If only the initial status of a person is reported, the variable would no longer be considered a status variable that changes over time, which simplifies anonymization. The disadvantage is that we can no longer see the progress, for example, in the person’s occupational and educational level over time.

If only the first and last statuses of a person in a record are reported, all events in between must either be deleted or replaced by the first stage or the last status.

Another very strict alternative would be to delete the link of a person from one year to the other; that is, for each person, another ID is provided from one year to another. However, this makes a longitudinal analysis difficult; thus, the data utility would suffer significantly.

Postrandomization could be an option, although the order and consistency of educational and occupational levels are either lost or biased to higher levels. For example, it makes no sense to lower a person’s education level over time; therefore, with realistic swapping probabilities in the PRAM, the education level would randomly increase but never decrease.

Another approach would be to apply traditional anonymization methods to patterns or subsets of the data, whereby individuals with the same pattern of event occurrence are considered as a subset to be anonymized. For example, the 2 individuals in Table 1 do not have the same pattern as they have a different number of events. This approach leads to a potentially large oversuppression but reduces the disclosure risk heavily. Studies aimed at analyzing the education and occupation of individuals over time might be possible, especially when data analysts impute the suppressed information.

Before deciding on one of these or even other alternative approaches, one has to think about the disclosure scenario. How likely is it that an attacker can merge their database with the anonymized data set provided to match and identify individuals? How likely is a nosy neighbor scenario and to what extent?

For an archive scenario, the following assumptions regarding the attacker’s knowledge are made:

- Only the last status of education of a person is known to the attacker, assuming that the attacker’s database is more or less an up-to-date archive containing the current educational level of a person used for matching. Here, it is neglected that the attacker has access to the historical sociodemographic status data of individuals.
- Only the last occupational status is known by an attacker, provided that the attacker’s database is more or less an up-to-date archive containing the current profession of a person used for matching.
- The attacker has knowledge of the static variables of sex and birth date.
- The attacker does not know the reason for in- and out-migration but knows the birth date, the start date, and the stop date.

For a nosy neighbor scenario, the following assumptions about the attacker’s knowledge are made:

- The (changing status) of the education of a person is known to the attacker over time, assuming that the attacker has individual knowledge of the historical development of the educational and occupational levels of a few individuals.
- The attacker has knowledge of the static variables of sex and birth date.
- The attacker may know the reason for in- and out-migration for certain individuals and the corresponding event time, and they may have knowledge about the birth date of certain individuals.

As the data go public as an open-access data set, a nosy neighbor scenario is possible and, thus, in focus. Therefore, we use the approach in which only the first and last observed statuses of a person are reported. This is a solution in which the change in a person’s status is reported without their intermediate improvements, whereas local suppression results in a low number of suppressions as not all stages are reported.

**Table 1:** Toy data set supporting a simple explanation to the problem to deal with time-varying information on status variables.

Person ID	(Event) year	Occupation	Education level
1	2010	2	2
1	2011	2	2
1	2012	3	2
1	2013	3	2
1	2014	3	2
1	2015	3	3
1	2016	4	3
2	2010	2	2
2	2015	3	3
2	2016	3	3

### Handling Event History Dates

#### General Considerations

To prevent (exact) record linkage and closest distance–based neighborhood matching, we suggest adding random noise to the event dates. An adequate obvious choice is to add approximately 100 days randomly. This prevents an attacker from successfully applying record linkage and is likely to prevent distance-based matching.

However, care must be taken to ensure that the order of events is maintained. For example, if a person has a birth date of May 15, 2009, and we hypothetically assume that this person out-migrated on June 5, 2009, in-migrated on July 6, and died on August 1, 2009, then a random noise of +40 or –40 to +60 or –60 days will completely upset the event order.

Thus, we need to modify the event data by adding or subtracting a sufficient number of days so that the individual cannot be identified, although the data utility and event order of the data are retained. More specifically, the addition of noise must be performed with the following constraints: (1) the order of events must be maintained; (2) the time span between events should remain the same as much as possible, naturally fulfilled by adding noise; (3) attacks with record linkage should not be successful; and (4) the number of events per person should remain unchanged.

This leads to a sequential approach that adds noise for each person, event by event, under certain restrictions, explained in more detail in the following paragraphs. Of course, the main parameter—the level of noise—must be determined on a use case and data set–specific basis.

#### Add Noise to One Event Date

**Figure 1:**
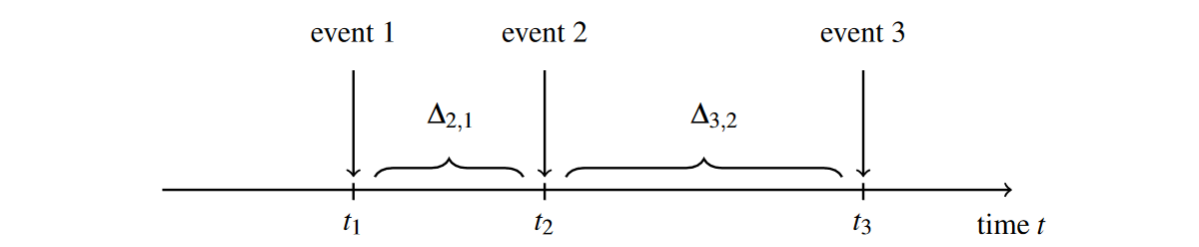
Schematic overview of 3 event history dates for one person and corresponding time span between the events.

For simplicity, equation 1 shows the case for 3 events, whereby noise is added for 1 person for event 2. Figure 1 shows this case with 3 event dates t_1_, t_2_, and t_3_, and the time span between events 1 and 2 (∆_2;1_) and events 2 and 3 (∆_3;2_).

It should be noted that extension to any number of events per person is possible and straightforward to implement, although the notation becomes more complicated.

With *s*, a Bernoulli random values∈{–1, 1} with *P*=.50 for random addition or subtraction of the event date, and u ~ U[ɛ_min_; ɛ_max_], which controls the number of noise (in days), a new (anonymized) event date t_2_^*^ is calculated using the following:


t_2_^*^ = t_2_ + u · s , if ∆_2,1_ > ɛ_max_ ∧ ∆_3,2_ > ɛ_max_
t_2_^*^ = t_2_ + u , if ∆_2,1_ ≤ ɛ_max_ ∧ ∆_3,2_ > ɛ_max_
t_2_^*^ = t_2_ – u , if ∆_2,1_ > ɛ_max_ ∧ ∆_3,2_ ≤ ɛ_max_
t_2_^*^ = t_2_ – u – (∆_2,1_ – 1) , if ∆_2,1_ > ɛ_max_ ∧ ∆_3,2_ ≤ ɛ_max_ ∧ min(∆_2,1_, ∆_3,2_) = ∆_2,1_

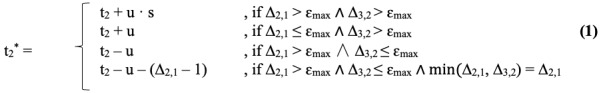



This ensures that the event order is preserved for t_1_, t_2_, and t_3_. Except for the first case, restrictions were applied as the distance between event data was smaller than the specified minimum noise range.

An alternative noise addition method is to draw u ~ N(µ, σ^2^) and round it to the next integer value.

#### Add Noise Sequentially Event by Event

The extension of equation 1 to all events of a person is achieved by the sequential application of noise to each event of a person. First, all recorded data of one person are stored, and the number of events of this individual, as well as the distance between all events, are recorded. For the first event, date t_1_ noise is either randomly subtracted or added; more precisely, it is subtracted without any restrictions and added less than the distance to the second event. Subsequently, for all other events recorded, in an additional loop considering one event date at the time, noise is added, as described above (equation 1) according to a predefined noise level (see *Disclosure Risk* and *Data Utility* section for further discussion on the level of noise). Therefore, first, for t_1_, noise is added leading to t_1_^*^, and then, noise is added to t_2_, considering possible restrictions from t_3_ and t_1_^*^ to not change the event order. Subsequently, noise is added to t_3_ considering t_2_^*^ and t_4_,..., until the last event date. Using this sequential approach, preservation of the event order is guaranteed.

Restrictions may occur if 3 consecutive events are very close to each other. If the maximal noise of the respective noise level is larger than the difference between t_2_^*^ and t_3_ and t_3_ and t_4_, it proceeds as follows. If the minimum of the event difference min(∆_2;1_;∆_3;2_) is larger than the predefined minimum noise, then take minimum=minimum noise and maximum noise=∆_2;1_ and ∆_3;2_, respectively, and sample at random. If the minimum of event difference min(∆_2;1_;∆_3;2_) is smaller than the minimum noise, then sample from a univariate distribution *U*(0; ∆_2;1_); same with ∆_3;2_ in the respective sampling direction as maximum or minimum noise. In the case of normal distribution while (noise < ∆_2;1_ ∧ noise > ∆_3;2_), draw a new value from *N*(μ=0; σ=50) until a valid noise is obtained.

Furthermore, we would like to briefly point out that it is necessary to consider the special data structure. It has already been mentioned that the event history dates cannot ideally be represented in columns, as there are different numbers of events and different events per person. Therefore, a separate row for each event in the data set is used to store the event code and date for a person; that is, individuals are represented in multiple rows. If a person was born within the observation period, he or she has an additional entry as an event in addition to the actual date of birth. Thus, if no birth date is registered under event dates, as the individual was born before data collection, then only one number is randomly added to the date of birth of a person in all rows of this person. If birth is also represented as event date information, the same noise (used to noise the event date on birth) has to be taken as for the column holding the birth date of the person; that is, the information on birth date and the event birth date is linked and must be considered adequately and consistently.

In the *Results* section, the noise level chosen for the HDSS core data set is presented, and further insights into the choice of noise level are provided.

### Putting It All Together

The event data are particularly important as they are numerical information that can be used for record linkage if the attacker has a database of exact event data. However, an attacker might only know the year of birth and death and then use this information for matching. In addition to the event history dates, variables with varying statuses over time must also be considered. Therefore, the changes in education and occupational levels are limited by indicating only the first and last status (Textbox 1).

For certain studies, for example, on fertility by educational level, the full history of event dates and changes in the educational level is needed. This is also true for various studies on the occupational level of individuals over time (eg, answering the question of whether well-educated individuals change their occupational levels quicker). In this case, the entire history of event data might be needed, and the previous procedure has to be adapted, in this case, for example, by anonymizing the patterns, as outlined previously.

**Steps of putting it together.**

**Step 1**

- Add random noise to event dates for each person sequentially, as described in the *Handling Event History Dates* section. This prevents record linkage and nearest-neighbor matching with an external database containing exact event dates and preserves the order of events.

**Step 2**

- Aggregate data (ie, from long to wide representation, where each line represents a person) so that each row contains the information of a person for the static variables (such as sex and birth date), first and latest education, and first and latest occupation and build new variables containing the year of birth, year of death, and number of events of a person.

**Step 3**

- Perform k-anonymity using local suppression using the implemented methods in sdcMicro [7] using the variables mentioned in step 2 to avoid uniques and prevent successful matching. If the year of the earliest or latest event or the year of birth is suppressed, the noised year and noised event date should also be suppressed. It should be noted that this was hardly the case as the importance was set such that the year of birth, year of death, and number of events of a person are the most important variables; thus, the suppression algorithm uses the remaining variables to make local suppressions.

**Step 4**

- Disaggregate the anonymized aggregated data (from wide to long representation, where each line represents an event). The data set now includes only the anonymized information on sex and the earliest and latest occupational and educational codes of a person.

### Estimation of the Disclosure Risk

The theory for estimating disclosure risk in a cross-sectional data set is well implemented, for example, in the R package sdcMicro [6,7]. In fact, for survey sample data, the approach of Franconi et al [38] or, for example, Skinner et al [27,39] can be used, or, for population data, the concepts of k-anonymity [40,41] or sample uniqueness [35,36]. We introduce an extension of this theory that provides a practical tool for quantifying disclosure risk for event history data.

Typically in anonymization, methods differ when continuous or categorical information is anonymized [6]. In addition, we distinguish between 2 scenarios—the matching of event dates (continuous measurements) and an attack on categorical key variables.

Event data are considered continuous measurements as there are multiple records for each person on a time scale.

As k-anonymity is already ensured (step 3) and population data are used, there is no need to quantify the disclosure risk for categorical key variables.

For continuous event dates, a neighborhood distance-based approach is proposed. Neighborhood matching, as introduced here and further introduced and applied in the *Results* section, assumes that the attacker has a database with exact event dates, which represents a worst-case scenario. For each individual in the anonymized data set, the nearest 3 individuals in the original nonanonymized data are determined by using Euclidean distances between event dates in the original and anonymized files. This is performed with replacement, meaning that the nearest neighbors are available to match for another individual in the data set. In case 1 of the 3 nearest neighbors is the correct match, we identify this observation to be of high risk. The number of risky observations is reported. The *Results* section shows the specific settings for our application.

## Results

### Anonymization of the Karonga HDSS Core Residency Data Set

First, it should be noted that the data set obviously cannot be spread into columns of events as migration and other event codes have possibly >1 entry, and the number of events differs between individuals. This makes it difficult to anonymize the data as the individuals have different events and different numbers of the same events at different times.

The key (identifying) variables are listed in Table 2.

Experiments with the HDSS core residency data set have shown that an additional identifying variable, the ID of the mother of a child, ID of the father and of the household, and the reason for in-migration and out-migration (reasons are marriage, divorce, start or end of work or education, and others) could potentially enlarge possible matches to approximately 10% of the original possible matches or individuals. Polygamy identifiers are not considered in this study. The usual approach for handling cluster information (eg, persons in households) for risk estimation of (enlarged) risk is, for example, described in Templ et al [6] and implemented in sdcMicro under the term of hierarchical risk estimation. However, as no further household information is available in this data set, this approach can be neglected. This is because household information can be used to identify individuals more easily; however, such additional household information is not available in our data set.

Other socioeconomic or sensible variables (eg, health status) were not included in the open-access data set.

**Table 2:** Key (identifying) variables of the health and demographic surveillance system core residency data set.

Key variable	Kind
Biological sex	Static variable
Year of birth	Static variable
Year of death	Static variable
Exact event date	Core event date^a^
Education	Status variable
Occupation	Status variable
Number of events per person	Static variable

^a^Contains dates at which the observed core events occurred (birth, death, in-migration, or out-migration).

### Anonymization of Event Dates (Details Related to Step 1)

According to the random principle, a drawn number of days is randomly added to or subtracted from the event dates of birth, death, in-migration, and out-migration (equation 1; *Add Noise to One Event Date* section).

Four levels of noise were considered. In 3 scenarios, integer numbers (noise in days denoted by ε) for each event of a person (with E being the number of events of a person) were drawn with equal probability from the following intervals—depending on the noise level. In addition, a fourth scenario with normally distributed random noise is considered:

1. Noise level 1: ε_min_=46; ε_max_=62
2. Noise level 2: ε_min_=76; ε_max_=93
3. Noise level 3: ε_min_=106; ε_max_=124
4. Noise level 4: u ~ N(µ=0; σ=50)

As described previously, random noise is added sequentially to the birth date, in-migration and out-migration dates, and death date to prevent record linkage and nearest-neighbor matching, with an external database containing exact event dates and information on sex, number of events, year of birth, year of death, occupational status, and educational level.

### Anonymization of Static and Status Key Variables (Details to Steps 2 to 3)

To prevent successful matching, we achieved 3-anonymity through global recoding and local suppression using the heuristic implemented in the R package sdcMicro [6,7].

New variables are built for the year of birth, year of death, and year of the first change of educational and occupational status and used as key variables along with the sex of a person and the number of events of a person. Intermediate changes in educational and occupational levels are dropped. K-anonymity is then achieved by local suppression using the implemented methods in sdcMicro [7]. If the year of the latest event or the year of birth is suppressed, the noised year and noised event date are also suppressed. The number of events and the year of birth and death are set to the highest importance so that the implemented (weighted) local suppression algorithm in Templ et al [7] likely does not include missing values in these variables. Note that one suppression in a variable with high importance would increase the loss (function) in utility for >1 suppression in a variable with low importance (see Templ et al [7] for details).

After event date anonymization and status variable anonymization, the data are again matched to transform them into their original shape.

### Disclosure Risk

To assess whether a data set was successfully anonymized, we quantified the disclosure risk. It must be reported only for event dates as, for the categorical key variables, k-anonymity is achieved, which satisfies our need to prevent successful matching.

The disclosure risk is calculated by matching each individual of the raw data set with the 3 nearest neighbors of the anonymized data with replacement using distance-based matching. In addition, an individual is matched with individuals who are born, died, or migrated within plus minus the same year as the true match, respectively, having the same (final) education, the same (final) occupation, and the same sex. If an individual has a missing value for one of these variables because of local suppression, that person is still considered a possible match if the rest of the variables meet the requirement.

If the match is correct, we assume that the attack was successful, and an individual can be reidentified. This means that if a person is in 3 of the nearest distances, we consider it unsafe. False-positive matches are not taken into account.

Table 3 reports the absolute and relative disclosure risk (in percentage) of the anonymized Karonga data set for all 4 scenarios, considering only individuals as possible matches who were born or had died or migrated in the range of +1 or –1 year of the date of birth, death, or migration, respectively, of the real match. We can observe that the risk is very low and that an attacker can hardly reidentify individuals. Note that the disclosure risk is already based on a worst-case scenario with 3 neighbors and by assuming the attacker uses the original nonanonymized data for matching. The low risk can also be explained by the fact that we choose ε_min_ to be relatively large; for example, for noise level 1 it is 46, meaning that for each event, the date is changed within at least 46 days. However, for death and birth, the risk increases as death is more unique than any of the other variables. The highest risk is connected with normal noise.

The computation time for neighborhood-based risk measurement, as proposed here, is high, and an implementation that uses parallel computing is preferable. Currently, the anonymization runs for 4 hours on a single-core Intel(R) Core i7-6700HQ central processing unit (CPU) with 2.60 GHz, and 8 days are spent for the risk assessment on all 4 noise levels on the HDSS core residency data set using 32 CPUs, Intel Xeon(R) Gold 5218 CPU with 2.30 GHz.

**Table 3:** Counts on successfully matched individuals and relative disclosure risk (in percentage; number of risky individuals divided by the number of individuals times 100) of the anonymized Karonga data set for all 4 levels of noises based on the matching scenario.

Scenario		Birth (number of successful matches)	Death (number of successful matches)	IMG^a^ (number of successful matches)	OMG^b^ (number of successful matches)
**Absolute risk**					
	*U*(46;62)	1669	177	220	394
	*U*(76;93)	1452	154	222	388
	*U*(106;124)	1271	151	178	383
	*N*(μ=0; σ=50)	1513	619	197	242
**Relative risk (%)**					
	U(46;62)	2.3	5.0	0.5	0.8
	U(76;93)	2.0	4.3	0.5	0.8
	U(106;124)	1.7	4.2	0.4	0.8
	*N*(μ=0; σ=50)	2.1	17.3	0.4	0.5

^a^IMG: in-migration.

^b^OMG: out-migration.

### Utility

Utility measures specialized in a particular field should always be preferred to general measures ([42]; eg, as implemented in sdcMicro). To check the data utility after anonymization, visual comparisons of the original nonanonymized and anonymized data sets, as well as chi-square tests comparing contingency tables obtained from original and anonymized data, are shown.

**Figure 2:**
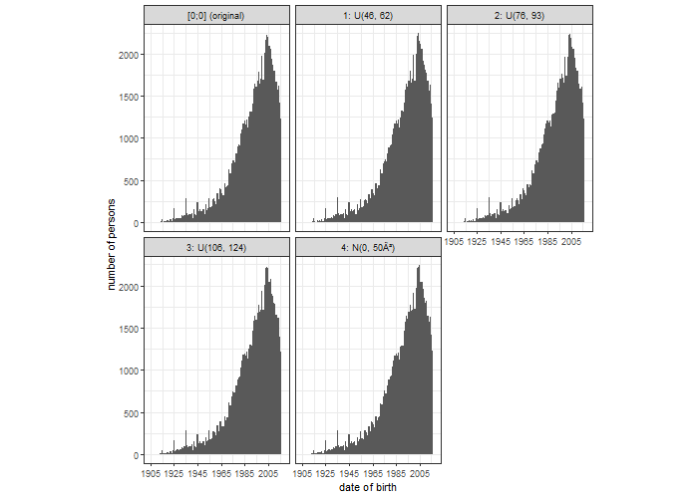
Distribution of the date of birth of the original data set and for the anonymized data set according to noise levels 1, 2, 3, and 4.

Figure 2 shows the distribution of the date of birth from the original data and the noised data sets. The original data show a heaping in 1925, 1937, and 1945, which is still visible in the modified versions of the data set. This is not surprising as the noise was not too large.

**Figure 3:**
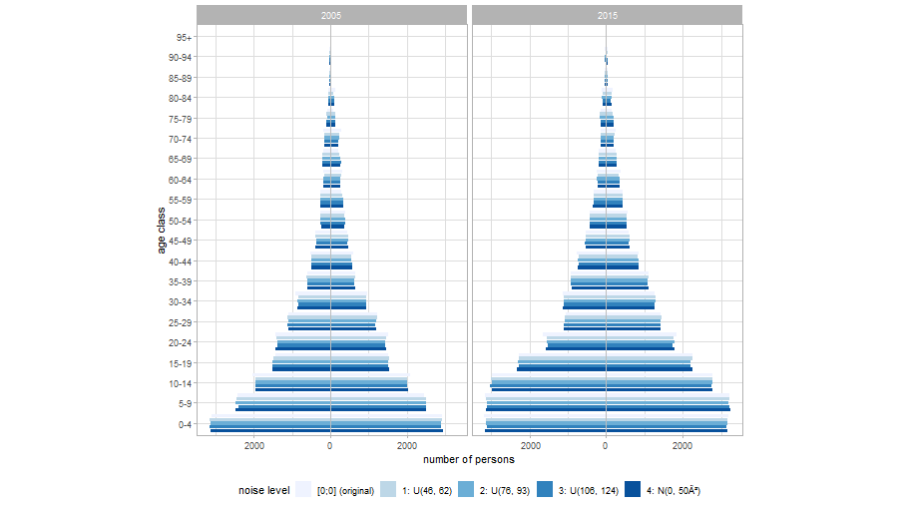
Population pyramids for 2005 and 2015 midyear population and age structure of the original and anonymized data according to noise levels 1, 2, 3, and 4 for men (left bars) and women (right bars).

The 2 midyear population pyramids for 2005 and 2015 are depicted in Figure 3. We distinguish between the population pyramids for the original nonanonymized data and anonymized data with noise levels of 1 to 4. Almost no differences were observed.

We do not explicitly show further graphs on the distribution of the date of death, in-migration, and out-migration, as the results are very similar to the previous figures; that is, there are no significant differences in the distributions.

Table 4 shows summary statistics of the time span between in-migration and subsequent out-migration of individuals. It shows only minimal differences; that is, all statistics are well preserved. The best results are obtained with noise scenario 4 (normal distributed noise). The results for out- to in-migration are comparable, except for the time between out- to in-migration. This can be shown in more details by a visualization.

**Figure 4:**
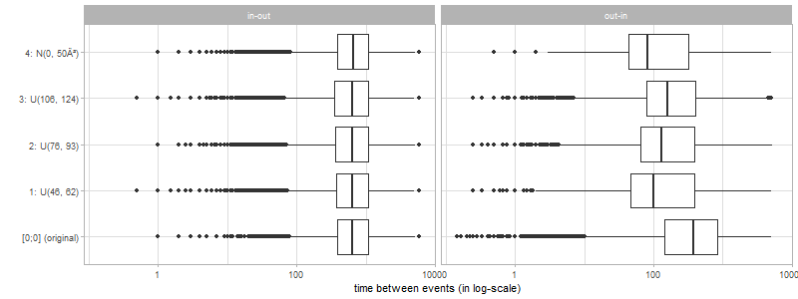
Time span (in log10 scale) between in-migration and subsequent out-migration and out-migration to subsequent in-migration of the original data set and for the anonymized data sets by noise levels 1, 2, 3, and 4. Regarding in-migration to out-migration and out-migration to in-migration only individuals who in- or out-migrate, respectively, are considered.

Figure 4 visualizes this time span between in-migration and subsequent out-migration, as well as between out-migration and in-migration by box plots. The x-axis is presented on a log_10_ scale to better see minimal differences in the distribution of the time span between the original nonanonymized data and the anonymized data (almost no differences can be seen in the original scale). Almost no differences were found in the time span for in-migration to out-migration.

For the number of days between the out-migration and in-migration of a person, the worst results were obtained by scenario 4 (normal distributed noise). The reason for this difference between in- and out-migration is that people tend to return after out-migration much earlier than they leave the place after in-migration. Normal noise tends to increase the number of days of consecutive events if the events are close together.

Table 5 presents the results of the statistical test. The cross-tabulation for age class×event code×sex×event time category (2000-2004, 2005-2009, 2010-2014, and 2015-2020) was calculated from the original nonanonymized data and for the anonymized data. The corresponding cell counts were compared with each other by using a chi-square test. The results of the chi-square tests (Table 5) showed that the null hypothesis of equality of anonymized and original data can never be rejected.

Naturally, the differences between original and anonymization increase with an increasing level of noise, as can be seen in all the presented tables and visualizations of data utility. The best utility was achieved by adding normal noise (Table 5). However, even with noise level 3, the structure is well preserved, and the data utility is very high for all 4 noise levels investigated.

For the anonymization of the status variables on education and occupation, including sex, number of events of a person, year of birth, and year of death, a few values were suppressed to achieve 3-anonymity (Table 6). The highest number of suppressions is present in variable end education (last educational status of a person), with approximately 0.64% (3735/583,480) suppression. Overall, 0.14% (808/583,480) of values were suppressed.

**Figure 5:**
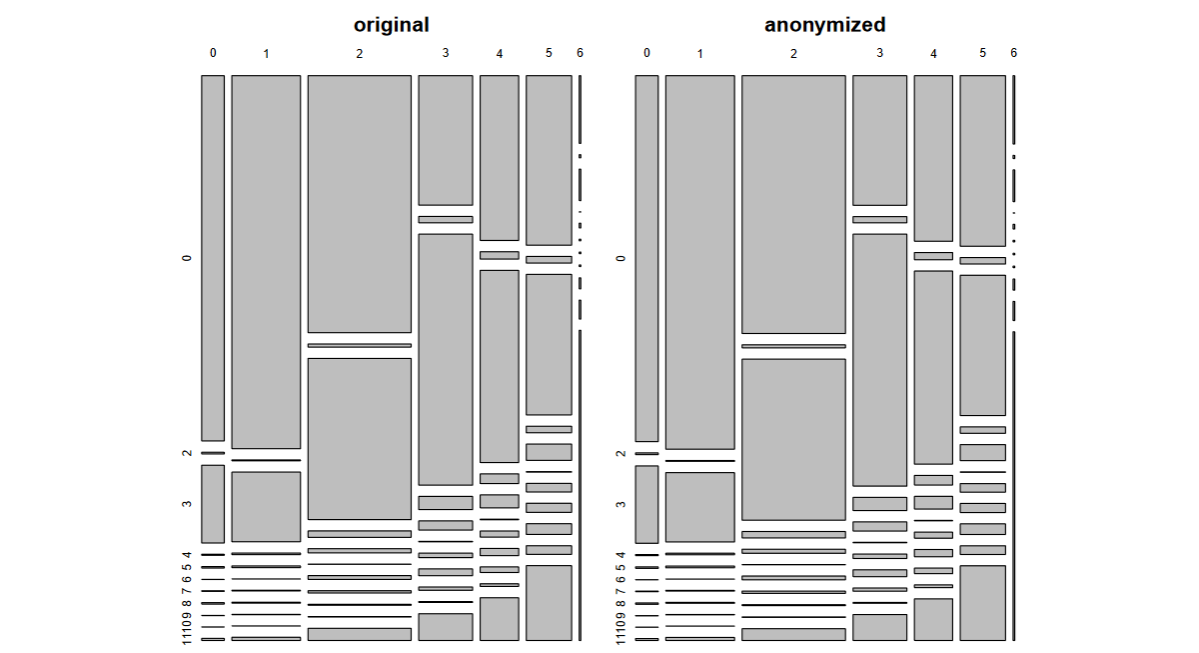
Relative frequencies of the latest educational and latest occupational status of individuals for the original and the anonymized data set.

For the static and status variables, one of the most important information might be the last status of occupation and education. Figure 5 shows the frequencies of the corresponding contingency tables. The differences were minimal and not detectable by visual comparison. This is even more true for the other tabulations.

**Table 4:** Summary statistics for the number of days between in-migration and subsequent out-migration of a person for noise levels 1 to 4.

Scenario	Values (minimum-maximum)	Values, mean (SD)	<100 days (%)
(0;0) (original)	(0-5909)	862.05 (714)	2.2
U(46;62)	(0-5805)	846.67 (716)	3.4
U(76;93)	(0-5832)	839.25 (717)	4.4
U(106;124)	(0-5906)	831.30 (720)	5.5
*N*(μ=0; σ=50)	(0-5859)	862.58 (716)	2.9

**Table 5:** Comparison of 4-dimensional contingency tables of the anonymized and original data using a chi-square test.

Statistics	U(46;62)	U(76;93)	U(106;124)	*N*(μ=0; σ=50)
Test statistic	46.08	73.58	121.39	37.52
Critical value	237.24	237.24	237.24	237.24
*P* value	.99	.99	.99	.99

**Table 6:** Percentage of suppressions per variable and total number of suppressions per variable.

Suppression	Sex	Base education	Base occupation	End education	End occupation	Number of events	Year of birth	Year of death
Suppressions (%)	0.03	0.22	0.07	0.64	0.13	0.02	0	0
Total suppressions	23	160	53	465	94	13	0	0

## Discussion

### Principal Findings

Providing open data (public use files) is a typical mechanism for HDSS data sharing, which is consistent with the funders’ [2] call for lowering barriers to data access and in the interest of implementing sustainable data sharing models. However, more stringent anonymization is required than that for access-restricted and contracted files used for scientific purposes.

Anonymizing HDSS data is challenging, and no easy-to-apply solutions are available. The details matter to ensure consistency or credibility, and context knowledge is key for successful implementation. The presented approach is novel in several respects. This is the first time that a systematic approach has been adopted to determine the anonymization requirements for residency data from LMIC HDSS studies or for any other longitudinal data generated in these settings. Previously, anonymization of HDSS data was performed on an ad hoc basis. We grouped the variables into static, status (time-varying), and core event–specific variables and tackled the anonymization relating to the variables in each of these groupings.

We achieved an anonymized data set with very low disclosure risk and high utility, ready for sharing as a public use data file.

Using distance-based neighborhood matching, we simulated an attack under a nosy neighbor situation and using the worst-case scenario, where attackers have full information on the original data. We showed that the risk of disclosure is very low, even when assuming the worst-case scenario.

We explicitly defined a procedure for anonymizing core event dates as a major part of the HDSS event history data anonymization. Different levels of noise addition to the event history dates were evaluated for disclosure risk and data utility. It was found that high utility was maintained, even with the highest level of noise. The basic properties of the event data such as order, time span, and number of events were preserved compared with the original data. As can be seen from the application and anonymization of event history dates, it is likely that the noise level and the loss of data utility will balance each other. Thus, a medium level of noise may be recommended to preserve the properties and usefulness of the data. In addition, the preservation of the time intervals between events is important for the successful implementation of this anonymization method. If the interval is too small, the added noise will is also automatically reduced by the algorithm.

Furthermore, our work explores the extent to which methods or tools such as sdcMicro can be used and for which aspects of longitudinal data. The guides for these tools focus on cross-sectional data and thus do not naturally lend themselves to the anonymization of multiple records per individual, which is the case in the Karonga HDSS core residency data that we used. In this regard, we transformed the time-varying variables of education level and occupation, year of death, year of birth, and the number of events for an individual before feeding them into the sdcMicro R package. The transformation involved limiting the number of transitions an individual had in the time-varying variables over time. This strategy preserves the data utility well, albeit providing fewer details than the original data.

The HDSS and medical science research communities in LMIC settings will be the primary beneficiaries of the results and methods presented in this paper; however, the results will be useful for anyone working on anonymizing longitudinal data sets, possibly including time-varying information and event history data with time-varying variables for purposes of sharing. If more sensitive variables such as medical conditions are added, l-diversity should also be checked. Alternatively, the PRAM [37] should be applied to medical conditions.

### Future Work

The proposed approach of combining the range of values for the status variables into a baseline value and a final value may not be optimal for some analyses. This is one of the realities of data anonymization; it almost always results in data of lower utility than the original data. Further work is required to explore alternative handling of the status variables to determine the optimal handling of the transitions in the time-varying variables.

The disclosure risk is calculated based on 3 nearest-neighbor distance-based matchings. This matching strategy is already quite complex, with some constraints described previously, as well as dealing with missing values. However, other matching strategies might be possible, and specialized record linkage software [43] might also be considered.

Further work is also required to determine the right amount of offset for the core event dates. To determine this, it might be important to gather data from the participants to estimate what it would take to sufficiently offset the dates so that the potential nosy neighbors are unable to make guesses even in cases where events such as in-migration are rare.

Of course, not all data sets might have exactly the same structure as the HDSS residency data set used here. Other longitudinal data sets from HDSS settings, such as those generated from the observation of tuberculosis episodes or sexual partnership episodes, may contain features not fully catered for by our approach here. These issues need to be explored further.

## Abbreviations

CPU: central processing unit
HDSS: health and demographic surveillance system
INSPIRE: Implementation Network for Sharing Population Information from Research Entities
LMIC: low- and middle-income countries
PRAM: postrandomization method
SDC: statistical disclosure control
